# Predicting sequelae and death after bacterial meningitis in childhood: A systematic review of prognostic studies

**DOI:** 10.1186/1471-2334-10-232

**Published:** 2010-08-05

**Authors:** Rogier CJ de Jonge, A Marceline van Furth, Merel Wassenaar, Reinoud JBJ Gemke, Caroline B Terwee

**Affiliations:** 1 VU University Medical Center, Department of Pediatrics and Infectious Diseases, Amsterdam, The Netherlands; 2 Emma Children's Hospital - Academic Medical Center, University of Amsterdam, Department of Neonatology, Amsterdam, The Netherlands; 3 VU University Medical Center, Department of Epidemiology and Biostatistics and the EMGO Institute for Health and Care Research, Amsterdam, the Netherlands

## Abstract

**Background:**

Bacterial meningitis (BM) is a severe infection responsible for high mortality and disabling sequelae. Early identification of patients at high risk of these outcomes is necessary to prevent their occurrence by adequate treatment as much as possible. For this reason, several prognostic models have been developed. The objective of this study is to summarize the evidence regarding prognostic factors predicting death or sequelae due to BM in children 0-18 years of age.

**Methods:**

A search in MEDLINE and EMBASE was conducted to identify prognostic studies on risk factors for mortality and sequelae after BM in children. Selection of abstracts, full-text articles and assessment of methodological quality using the QUIPS checklist was performed by two reviewers independently. Data on prognostic factors per outcome were summarized.

**Results:**

Of the 31 studies identified, 15 were of moderate to high quality. Due to substantial heterogeneity in study characteristics and evaluated prognostic factors, no quantitative analysis was performed. Prognostic factors found to be statistically significant in more than one study of moderate or high quality are: complaints >48 hours before admission, coma/impaired consciousness, (prolonged duration of) seizures, (prolonged) fever, shock, peripheral circulatory failure, respiratory distress, absence of petechiae, causative pathogen *Streptococcus pneumoniae*, young age, male gender, several cerebrospinal fluid (CSF) parameters and white blood cell (WBC) count.

**Conclusions:**

Although several important prognostic factors for the prediction of mortality or sequelae after BM were identified, the inability to perform a pooled analysis makes the exact (independent) predictive value of these factors uncertain. This emphasizes the need for additional well-conducted prognostic studies.

## Background

Bacterial meningitis (BM) is a severe infection of the central nervous system which occurs especially in children <5 years of age. Although the occurrence of negative consequences of BM in developed countries is strongly reduced by vaccination strategies, antibiotic treatment and good care facilities, BM is still responsible for substantial morbidity and mortality in both developing and developed countries [1-3].

The mortality rate is approximately 5%, and the long-term morbidity, mainly consisting of persistent neurological sequelae, is 15% [2,4-6]. Sensorineural hearing loss, seizures, motor problems, hydrocephalus and mental retardation [4,7-10], as well as more subtle outcomes like cognitive, academic and behavioral problems are observed in post-meningitis children [5,11].

In pediatric care, the goal must be to prevent these sequelae as much as possible. Therefore, early recognition of children with BM with high risk for the development of sequelae is mandatory [5,12-15]. For this reason, several studies have developed prediction models or have proposed prognostic factors for mortality or morbidity in children after BM [5-9,12-37]. The aim of the present study was to systematically review the available evidence regarding prognostic factors predicting death or sequelae due to BM in children aged 0-18 years in both developing and developed countries.

## Methods

### Literature selection

A systematic search of MEDLINE and EMBASE until March 20^th ^2009 was conducted to identify prognostic studies on mortality or various sequelae after BM in children. The search focused on BM using terms for the 10 most common causative pathogens according to the Netherlands Reference Laboratory for Bacterial Meningitis [38]. These pathogens are listed in Apendix 1. Tuberculoid meningitis or rare forms of BM were excluded. The search was refined using MeSH terms and text words on: morbidity, mortality, cause of death, survival rate, survival, prognos*, predict*, course*, cohort* longitudinal, cohort studies, follow-up, followup, follow up, follow-up studies. The search strategies used for Medline and Embase are included in Appendix 2. All abstracts found were screened by two reviewers independently (RdJ and MW). Those potentially eligible for inclusion were read in full text by the same two reviewers independently and subsequently discussed during a consensus meeting. Reference lists of each of the selected publications were checked to retrieve relevant publications which had not been identified by the computerized search.

The publications had to meet the following inclusion criteria, which were defined prior to the search:

- The study aimed to identify prognostic factors on mortality or various sequelae due to BM. Only studies designed as prognosis studies were included. Studies designed to analyze an associative model were excluded.

- The study was designed as a longitudinal cohort study, with at least one follow-up measurement. Both prospective and retrospective studies were included.

- BM had occurred at 0-18 years of age.

- Results were published in English as full report articles in international journals from January 1960 until March 20^th ^2009.

### Quality Assessment

The assessment of the methodological quality was performed using the Quality In Prognosis Studies (QUIPS) tool, designed for systematic reviews of prognostic studies through international expert consensus (Table 1) [39]. This assessment was performed independently by two authors (RdJ and MW). Disagreements between both authors were discussed during a consensus meeting.

**Table 1:** Used (adapted) QUIPS list for scoring methodological quality of prognosis studies

Criteria	Score		
	+	+/-	-
**1. Study participation**			
• Target population	3	1.5	0
• Sampling frame	3	1.5	0
• Inclusion criteria	3	1.5	0
• Baseline study population	3	1.5	0
• Adequate study participation	3	1.5	0

**2. Study attrition**			
• Proportion of population available for analysis	5	2.5	0
• Outcome and prognostic factor information on those lost to follow up	5	2.5	0
• Reasons and potential impact of subjects lost to follow up	5	2.5	0

**3. Measurement of prognostic factors**			
• Definition of prognostic factor	5	2.5	0
• Valid and reliable measurement of prognostic factor	5	2.5	0
• Method and setting of prognostic factor measurement	5	2.5	0

**4. Measurement of outcomes**			
• Definition of outcome	5	2.5	0
• Valid and reliable measurement of outcome	5	2.5	0
• Method and setting of outcome measurement	5	2.5	0

**5. Statistical analysis and presentation**			
• Presentation of analytical strategy	5	2.5	0
• Model development strategy	5	2.5	0
• Reporting of results	5	2.5	0

The QUIPS contains six categories assessing (1) bias due to patient selection, (2) attrition, (3) measurement of prognostic factors, (4) outcome measurement, (5) confounding on statistical analysis, and (6) confounding on presentation. The items on confounding were considered irrelevant for our study because in studies regarding prognosis, the design to predict a specific outcome based on a combination of several possible prognostic factors, confounding is not an issue. The remaining 17 items of the five categories were each scored to assess the quality of the included study. High quality ('+') was scored when there was low risk of bias, moderate quality ('+/-') with moderate risk, and low quality ('-') when there was high risk of bias.

To strengthen the discriminative capacity of the QUIPS we developed a scoring algorithm. All five categories were given a maximum of 15 points each, equally divided over all items per category. For all items we assigned 5 points in case of low risk of bias and 2.5 and 0 in case of moderate and high risk of bias, respectively. Except for category 1 (patient selection bias) which contained five instead of three items. Here we assigned 3 points in case of low risk of bias and 1.5 and 0 in case of moderate and high risk of bias, respectively. A total score, with a maximum of 75 points, was calculated by summing up the scores per item. A priori, we chose to consider ≥60 points (≥80% of the maximum attainable score) as high quality, between 45 and 60 points (≥60% of the maximum attainable score) as moderate/high quality and <45 points as low quality studies.

### Data extraction and analysis

Of the selected studies, data were extracted regarding study population (age at infection, country), causative pathogen, design (prospective or retrospective), duration of follow-up, method of analysis (uni- or multivariate), outcome measures and independent statistically significant prognostic factors from multivariate analysis or, if not available, from univariate analysis (p < 0.05). To facilitate interpretation and comparison of the results, data were categorized per outcome: (1) hearing loss, (2) mortality, (3) neurological sequelae, or (4) poor outcome when the original study made no distinction between mortality and neurological sequelae. Both short- and long-term outcomes were included.

This review did not aim to analyze original study data, therefore only the data presented in the manuscripts was used. Authors were not approached for insight in their data.

### Analysis of prognostic factors

Due to heterogeneity in study design, study population and analyses of the included studies, no quantitative analysis was performed. Instead, the prognostic factors predictive for mortality or sequelae after BM were summarized per outcome category. Prognostic factors reported in different papers on the same cohort were counted once. Due to the large variety in proposed factors found, only those factors found significant (p < 0.05) in more than one study of moderate/high quality were presented.

## Results

### Selection of studies

**Figure 1:**
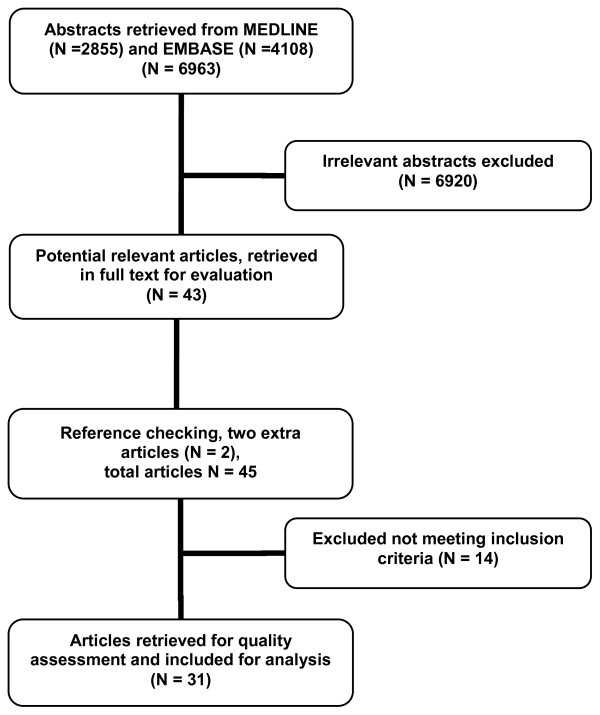
**Selection and number of publications**.

Figure 1 presents a flow chart of the study selection. The search strategy yielded 6,963 citations. Of these, 43 articles seemed to fulfill the inclusion criteria and were retrieved in full text. Two additional articles were identified by checking the reference lists. Review of these 45 articles resulted in exclusion of 14 articles not meeting the inclusion criteria. Eleven studies were excluded based on design (one letter, one validation study and nine presenting an association model instead of a prognostic model), one study dealt with diagnosis (prediction of meningitis instead of sequelae), and two studies were excluded because no differentiation was made between viral or aseptic and BM for outcome measurement. Finally, 31 articles were included and assessed on methodological quality.

### Methodological quality

The results of the quality assessment are presented in Table 2. The overall quality score ranged from 17 to 62.5 points with a median score of 43.5. Based on our cutoff of ≥60 and ≥45 points, respectively, one article was classified as high quality, 14 articles were classified as moderate/high quality and 16 articles as low quality studies.

**Table 2:** Results of quality assessment of studies on mortality or sequelae after bacterial meningitis.

Study	Study participation	Study attrition	Measurement of prognostic factors	Measurement of outcomes	Statistical analysis and presentation	Quality score (points)	Quality: + = high +/- = moderate - = low
Koomen et al., 2004 [5]	15	12.5	10	12.5	12.5	62.5	+

Lovera et al., 2005 [33]	13.5	7.5	12.5	10	12.5	56	+/-
Roine et al., 2008 [35]	15	10	7.5	7.5	15	55	+/-
Oostenbrink et al., 2002 [21]	15	7.5	10	10	12.5	55	+/-
Pelkonen et al., 2009 [6]	12	10	12.5	7.5	12.5	54.5	+/-
Forsyth et al., 2004 [18]	10.5	10	7.5	15	10	53	+/-
Biesheuvel et al., 2006 [24]	12	7.5	10	10	12.5	52	+/-
Pagliano et al., 2007 [19]	12	5	12.5	10	12.5	52	+/-
Koomen et al., 2003 [7]	15	7.5	10	7.5	10	50	+/-
Woolley et al., 1999 [9]	12	5	7.5	12.5	12.5	49.5	+/-
Klinger et al., 2000 [31]	13.5	7.5	7.5	10	10	48.5	+/-
Singhi et al., 2007 [15]	13.5	5	5	12.5	12.5	48.5	+/-
Kornelisse et al., 1995 [8]	10.5	5	10	10	12.5	48	+/-
Fakhir et al., 1992 [27]	12	7.5	7.5	12.5	7.5	47	+/-
Akpede et al., 1999 [16]	10.5	7.5	10	10	7.5	45.5	+/-

Kaaresen et al., 1995 [29]	13.5	7.5	7.5	5	10	43.5	-
Kutz et al., 2006 [14]	13.5	5	7.5	7.5	10	43.5	-
Pikis et al., 1996 [20]	13.5	7.5	7.5	7.5	7.5	43.5	-
Pomeroy et al., 1990 [34]	15	5	5	10	7.5	42.5	-
Wasier et al., 2005 [37]	10.5	5	7.5	7.5	10	40.5	-
Grimwood et al., 1996 [12]	7.5	5	7.5	12.5	7.5	40	-
Edwards et al., 1985 [17]	10.5	7.5	7.5	10	2.5	38	-
Letson et al., 1992 [32]	10.5	5	5	10	7.5	38	-
Chao et al., 2008 [26]	12	2.5	5	7.5	5	32	-
Johnson et al. 2007 [28]	12	2.5	5	7.5	5	32	-
Bortolussi et al., 1978 [25]	10.5	5	2.5	7.5	5	30.5	-
Antilla et al., 1994 [22]	7.5	2.5	7.5	7.5	2.5	27.5	-
Kirimi et al., 2003 [30]	7.5	5	7.5	2.5	5	27.5	-
Valmari et al., 1987 [36]	4.5	2.5	5	5	7.5	24.5	-
Herson et al., 1977 [13]	4.5	2.5	5	2.5	5	19.5	-
Bhat et al., 1987 [23]	4.5	2.5	2.5	2.5	5	17	-

Studies of moderate/high quality scored well on patient selection, outcome measurement, statistical analysis and presentation, and relatively well on prognostic factor measurement. However, many moderate/high quality studies scored poor on attrition. Studies classified as low quality scored relatively well on patient selection, but poor on all other categories. A poor score on prognostic factor measurement was often due to the fact the studies did not mention all factors considered in their analysis but presented only those factors found significant.

### Study characteristics

Table 3 summarizes the study characteristics of all included publications. Studies were grouped by outcome categories and ranked by quality. Of all 31 included studies, four studies focused on hearing loss, four on mortality, five on neurological sequelae ranging from mild to severe, and another 12 studies focused on poor outcome. The remaining six studies focused on both mortality and neurological sequelae, for which results were presented separately. Therefore, these studies were included more than once. The majority of all studies (n = 21) had a retrospective study design and 22 were conducted in developed countries. Sixteen studies performed a multivariate analysis. Although Klinger *et al*. [31] performed a multivariate analysis, we reported the prognostic factors based on their univariate analysis, since this study reported several models for different time intervals which was more difficult to compare with other results. There was considerable variation among studies with respect to population size (37 - 716 patients) and follow-up duration (from moment of hospital discharge up to 23 years later). Studies also varied with respect to age at infection (0-17, of which three studies considered specifically neonatal/infant age) and type of causative pathogen studied (varying from describing all types (n = 17), to those only studying a specific microorganism (n = 9; mainly *Streptococcus pneumoniae*)) or more than one but not all microorganisms (n = 5; mainly concerning all microorganisms; however, excluding *Haemophilus influenzae type B (HiB))*.

**Table 3:** Study characteristics of studies on prediction of sequelae after bacterial meningitis.

Study	Score (quality)	Design	Developed or developing (Country)	N	Age at infection	Pathogen	Follow-up duration	Outcome:	Analysis	Significant prognostic factors From multivariate analysis Or from univariate analysis with p < 0.05
Forsyth et al., 2004 [18]	53 (+/-)	Prospective	Developing (Malawi)	343	2 months - 13 yr	All	1 and 6 months after discharge	Hearing loss	Univariate	Coma, positive CSF Gram stain, a low peripheral WBC count, high CSF protein level, associated neurological sequelae
Koomen et al., 2003 [7]	50 (+/-)	Retrospective	Developed (The Netherlands)	628	0 - 9.5 yr (mean 2.4yr)	Non Hib		Hearing loss	Multivariate	History of symptoms >2 days, absence petechiae, low CSF glucose level, causative pathogen (*S. pneumoniae*), ataxia
Woolley et al., 1999 [9]	49,5 (+/-)	Retrospective	Developed (UK)	432	Median 7.7 months	All	6 months intervals for at least 1 yr (range 1-5 yr)	Hearing loss	Multivariate	Male sex, increased ICP, low CSF glucose level, causative pathogen (*S. pneumoniae*), presence nuchal rigidity
Kutz et al., 2006 [14]	43,5 (-)	Retrospective	Developed (USA)	171	3 months 17 yr (mean 3.8 yr)	All	During hospitalization (longer if necessary)	Hearing loss	Univariate	Long duration hospitalization, cranial nerve neuropathy, low CSF glucose level, high CSF protein level, seizures (not significant in case of *S. pneumoniae*)

Lovera et al., 2005 [33]	56 (+/-)	Retrospective	Developing (Paraguay)	72	35 days - 15 yr (mean 48 months)	S. pneumoniae	During hospitalization	Mortality	Univariate	Age <12 months, coma, seizures, prolonged duration of seizures >48 h, low CSF WBC count, high CSF protein (albumin) level, low CSF glucose level, low peripheral WBC count, low Hb
Roine et al., 2008 [35]	55 (+/-)	Prospective	Developing (6 countries in Latin America)	654 total cohort, 332 included in analysis	Median 8 months in patients who died, median 12 months in survivors (not otherwise reported)	all	During hospitalization	Mortality	Multivariate	Impaired consciousness, poor peripheral circulation, high CSF protein level
Pelkonen et al., 2009 [6]	54,5 (+/-)	Retrospective	Developing (Angola)	403 total cohort, 290 included in analysis	Median 9.0 months	All	During hospitalization	Mortality	Multivariate	Impaired consciousness, severe dyspnea, convulsions during hospitalization
Kornelisse et al., 1995 [8]	48 (+/-)	Retrospective	Developed (The Netherlands)	83	3 days- 12.3 yr (median 8 months)	*S. pneumoniae*	During hospitalization	Mortality	Univariate	Comatose level of consciousness, shock, respiratory distress, low peripheral WBC count, low serum sodium level, high CSF protein level
Fakhir et al., 1992 [27]	47 (+/-)	Retrospective	Developing (India)	247	1 month - 14 yr	*N. meningitidis*	During hospitalization	Mortality	Univariate	Illness duration <12 h, hypotension, peripheral circulatory failure, coma (disturbed sensorium), rash duration <12 h, rash extent widespread, fever >40°C, absent neck rigidity, low peripheral WBC count, low ESR, low platelet count
Akpede et al., 1999 [16]	45,5 (+/-)	Prospective	Developing (Nigeria)	109	>1 month - 15 yr	All	During hospitalization (after discharge?)	Mortality	Univariate	Seizures, coma, shock
Wasier et al., 2005 [37]	40,5 (-)	Retrospective	Developed (France)	49	1 - 108 months (median 17 months)	*S. pneumoniae*	1-12 yr (mean 5 yr)	Mortality	Multivariate	High PRISM II score, low peripheral WBC count, low platelet count
Chao et al., 2008 [26]	32 (-)	Retrospective	Developing (Taiwan)	37	3 months - 11 yr (mean 37 months)	*S. pneumoniae*		Mortality	Univariate	Coma, shock, mechanical ventilation (endotracheal tube intubation), hyponatremia, low CSF WBC count, low CSF glucose level, low CSF/blood glucose ratio
Johnson et al., 2007 [28]	32 (-)	Retrospective	Developing (Nigeria)	71	<16 yr	All	During hospitalization	Mortality	Univariate	Respiratory distress, purulent/turbid CSF appearance, high CSF protein level, low CSF glucose level
Bortolussi et al., 1978 [25]	30,5 (-)	Retrospective	Developed (Canada)	52	Neonates (<1 month)	All	During hospitalization	Mortality	Univariate	low peripheral WBC count, thrombocytopenia, low birth weight <2500 g

**Study**	**Score (quality)**	**Design**	**Developed or developing (Country)**	**N**	**Age at infection**	**Pathogen**	**Follow-up duration**	**Outcome: sequelae**	**Analysis**	**Significant prognostic factors** **From multivariate analysis** **Or from univariate analysis with p < 0.05**

Koomen et al., 2004 [5]	62,5 (+)	Retrospective	Developed (The Netherlands)	182	0 - 9.5 yr (mean 2.4yr)	Non- Hib	4.0 - 10.4 yr post meningitis (average 7.4 yr)	Neurological sequelae Academic & behavioural limitations	Multivariate	male gender, low birth weight ≤3000 g, low educational level father, causative pathogen (*S. pneumoniae*), low CSF WBC count, delay >6 h start antibiotics, dexamethasone use ≤2 days, anticonvulsive treatment of seizures, prolonged fever >9 days
Pelkonen et al., 2009 [6]	54,5 (+/-)	Retrospective	Developing (Angola)	403 total cohort, 249 survivors, 200 included in analysys	Median 9.0 months	All	During hospitalization	Severe neurological sequelae	Multivariate	History of symptoms >3 days, impaired consciousness, convulsions during hospitalization
Biesheuvel et al., 2006 [24]	52 (+/-)	Retrospective	Developed (The Netherlands)	88 (derivation) 628 (validation)	0.9 - 5.8 yr (mean 2.8 yr) (derivation) and 0 - 9.5 yr (mean 1.9 yr) (validation)	Non Hib		Neurological sequelae Both mild and severe	Multivariate	Seizures (atypical convulsions), absence petechiae/ecchymoses, low body temperature <40°C, high body temperature/fever >40°, causative pathogen (*S. pneumoniae*), use of anti epileptic drugs >2 days
Singhi et al., 2007 [15]	48,5 (+/-)	Prospective	Developing (India)	80	2 months - 12 yr (mean 31.4 months)	All	12-44 months after discharge (mean 27.5 months)	Neurological sequelae Both mild and severe	Multivariate	coma, cranial nerve palsy, absent deep tendon reflexes
Kornelisse et al., 1995 [8]	48 (+/-)	Retrospective	Developed (The Netherlands)	83	3 days- 12.3 yr (median 8 months)	*S. pneumoniae*	Hospital duration	Neurological sequelae Both mild and severe	Univariate	High clinical severity score (Herson & Todd score), vomiting, shock, low peripheral WBC count, low CSF WBC count, low CSF glucose level
Akpede et al., 1999 [16]	45,5 (+/-)	Prospective	Developing (Nigeria)	109	>1 month - 15 yr	All	During hospitalization (after discharge?)	Neurological sequelae Both mild and severe motor and sensory sequelae	Univariate	Young age ≤2 yr, seizures, coma, prolonged fever >7 days, antibiotic treatment, focal nerve deficits, abnormal posturing, abnormal muscle tone
Pikis et al., 1996 [20]	43,5 (-)	Retrospective	Developed (Greece)	47	1 month - 14 yr (mean 2.6 yr)	*S. pneumoniae*	4- 23 yr (mean 12.4 yr)	Neurologic sequelae Both mild and severe	Multivariate	Coma, high peripheral WBC count
Pomeroy et al., 1990 [34]	42,5 (-)	Prospective	Developed (USA)	185	1 month - 14 yr (median 10 months)	All	1,3,6,12, months after discharge and yearly up to 6 yr	Neurologic sequelae Both mild and severe	Univariate	Seizures, low CSF glucose level
Chao et al., 2008 [26]	32 (-)	Retrospective	Developing (Taiwan)	37	3 months - 11 yr (mean 37 months)	*S. pneumoniae*		Neurological sequelae Both mild and severe lasting >6 months	Univariate	Focal neurological signs, seizures

**Study**	**Score (quality)**	**Design**	**Developed or developing (Country)**	**N**	**Age at infection**	**Pathogen**	**Follow-up duration**	**Outcome: poor outcome**	**Analysis**	**Significant prognostic factors** **From multivariate analysis** **Or from univariate analysis with p < 0.05**

Lovera et al., 2005 [33]	56 (+/-)	Retrospective	Developing (Paraguay)	72	35 days - 15 yr (mean 48 months)	*S. pneumoniae*	During hospitalization	Mortality & neurological sequelae	Univariate	Young age <12 months, coma, seizures, seizure duration >48 h, low CSF WBC count, high CSF protein (albumin) level, low CSF glucose level, low peripheral WBC count, low Hemoglobin
Oostenbrink et al., 2002 [21]	55 (+/-)	Retrospective	Developed (The Netherlands)	93	1 month - 15 yr (median 2.8 yr)	Non Hib	0.6 yr	Mortality & neurological sequelae	Multivariate	male gender, seizures (atypical convulsions), low body temperature, causative pathogen (*S. pneumoniae*)
Roine et al., 2008 [35]	55 (+/-)	Prospective	Developing (6 countries in Latin America)	642 total cohort, 356 included in analysis	Median 7 months in patients whith positive outcome measure died, median 14 months in patients without (not otherwise reported)	all	During hospitalization	Mortality & severe neurological sequelae	Multivariate	Impaired consciousness, history of symptoms >48 h, high CSF protein level, low peripheral WBC count
Roine et al., 2008 [35]	55 (+/-)	Prospective	Developing (6 countries in Latin America)	641 total cohort, 296 included in analysis	Median 7 months in patients whith positive outcome measure died, median 18 months in patients without (not otherwise reported)	all	During hospitalization	Mortality & all neurological sequelae	Multivariate	Impaired consciousness, convulsions before admission, poor peripheral circulation, low CSF glucose level, low peripheral WBC count
Pagliano et al., 2007 [19]	52 (+/-)	Prospective	Developed (Italy)	64	1 month -14 yr (median 26 months)	*S. pneumoniae*	8 weeks	Mortality & neurological sequelae	Multivariate	ICU admission, low CSF WBC count, penicillin nonsusceptibility

Klinger et al., 2000 [31]	48,5 (+/-)	Retrospective	Developed (Canada)	101	Neonates 1-28 days	All	1 yr	Mortality & neurological sequelae	Univariate	Hypotension, coma, inotrope, seizure duration >12 h, status epilepticus, low CSF/blood glucose level, low peripheral WBC count, abnormal neurological examination at discharge, ventilation, no. of anticonvulsants
Klinger et al., 2000 [31]	48,5 (+/-)	Retrospective	Developed (Canada)	101	Neonates 1-28 days	All	1 yr During hospitalization,	Mortality & neurological sequelae	*multivariate analysis	* For different time schedules during and after hospital admission not presented here
Akpede et al., 1999 [16]	45,5 (+/-)	Prospective	Developing (Nigeria)	109	>1 month - 15 yr	All	Possibly after discharge in neurologic clinic	Mortality & neurological sequelae	Univariate	Young age ≤2 yr, seizures, coma, shock, prolonged fever, >7 days antibiotic treatment, no meningeal signs, focal nerve deficits, abnormal posturing, abnormal muscle tone
Kaaresen et al., 1995 [29]	43,5 (-)	Retrospective	Developed (Norway)	92	Median 1.9 yr (range 1 month - 13.8 yr)	All	Hospital duration and mean 6 weeks afterwards, or longer if necessary	Mortality & neurological sequelae	Multivariate	History of symptoms >48 h, seizures, high body temperature,>38 °C, peripheral vasoconstriction, low CSF WBC count
Grimwood et al., 1996 [12]	40 (-)	Prospective	Developed (Australia)	138	3 months - 14 yr	All	Mean 6.7 yr post meningitis (range 5.3-9.3 yr)	Mortality & neurological sequelae	Multivariate	Young age ≤12 months, long symptom duration before diagnosis >24 h, seizures >72 h, focal neurological signs
Edwards et al., 1985 [17]	38 (-)	Retrospective	Developed (USA)	51	Infants (not further described)	Group B *streptococcus*	Mean 6 yr (range 3.3- 9.0 yr	Mortality & neurological sequelae	Univariate	Coma, hypotension (BP <40 mm Hg), low peripheral WBC count, low PMN, high CSF protein level
Letson et al., 1992 [32]	38 (-)	Retrospective	Developed (USA)	53	3.5 weeks - 30 months (mean 8 months	*H. influenzae b* *S. pneumoniae*	Mean 35 months	Mortality & neurological sequelae	Multivariate	Seizures, male gender, low CSF glucose level
Anttila et al., 1994 [22]	27,5 (-)	Prospective	Developed (Finland)	143	Mean 30 months range 3 months - 15 yr	All	During hospitalization, at discharge and at 2 weeks, 3,6,12 months	Mortality & neurological sequelae	Univariate	Low body temperature, coma, neck rigidity, leaving supine position
Kirimi et al., 2003 [30]	27,5 (-)	Prospective	Developing (Turkey)	48	2 months - 13 yr	all	Hospital duration	Mortality & neurological sequelae	Univariate	Fever >36-48 h after admission, coma 6-48 h after admission, anaemia, prolonged anaemia >3 days, low CSF WBC count, high CRP level, high CSF WBC count >3 days, low CRP level >3 days, antibiotic treatment
Valmari et al., 1987 [36]	24,5 (-)	Retrospective	Developed (Finland)	123 developing model 98 validation model	1 month- 14 yr mean 30 months developing model mean 20 months validation model	all	Mean 2 months	Mortality & neurological sequelae	Multivariate	Male sex, low CSF glucose level, low CSF WBC count, otitis media, low Hb, low serum potassium level, positive CSF gram stain, focal neurological signs, low peripheral WBC count, low CSF granulocyte %, low platelet count, neck rigidity, absence petechiae, duration of symptoms >48 h
Herson et al., 1977 [13]	19,5 (-)	Retrospective	Developed (USA)	73	6 weeks - 5 yr	H. influenzae b	Hospital duration, Residual morbidity: 3 months - 2 yr	Mortality & neurological sequelae	Univariate	Coma, low body temperature, seizures, shock (BP <60 mm Hg), young age <12 months, low CSF WBC count, low Hb, low CSF glucose level, prolonged symptom duration
Bhat et al., 1987 [23]	17 (-)	Prospective	Developing (India)	256	Non neonatal (not further described)	all		Mortality & neurological sequelae	Univariate	Duration of illness prior to therapy >7 days, low body temperature, coma, associated illness, low peripheral WBC count, purulent/turbid CSF appearance, high CSF WBC count, high CSF protein level, low CSF glucose level, neck rigidity, distension of abdomen, no meningeal signs, positive gram stain, positive culture, type of causative pathogen

Studies are grouped by outcome categories and ranked by quality.

### Prognostic factors

Table 4 summarizes the most important prognostic factors for sequelae and death after BM per type of outcome. For mortality and various sequelae together, 75 different possible prognostic factors were identified as significant by the included studies. Many of these factors might be of influence for the prediction of sequelae. However, it is implausible that all of them will be (equally) important. And due to poor study quality, factors not predictive for sequelae or death might have been found as prognostic factors. We therefore considered only those factors found significant in more than one study of moderate/high quality as evidence for being potentially important factors. Results from univariate and multivariate analyses are presented separately. Factors reported in studies of low quality are reported combined and not per type of outcome.

**Table 4:** Summary of prognostic factors.

Prognostic factor	Moderate/high quality studies with multivariate analysis				Moderate/high quality studies with univariate analysis				Low quality studies with multivariate analysis	Low quality studies with univariate analysis
	**Hearing loss**	**Mortality**	**Neurological sequelae**	**Poor outcome**	**Hearing loss**	**Mortality**	**Neurological sequelae**	**Poor outcome**	**All outcomes**	**All outcomes**

History of symptoms >48 h	1x			1x					1x	
Coma/impaired consciousness		2x	2x	2x	1x	4x	1x	3x	1x	6x
Seizures		1x	2x	2x		2x	1x	2x	2x	4x
Shock/hypotension						3x	1x	2x		3x
Peripheral circulatory failure		1x		1x		1x				1x
Severe respiratory distress		1x				1x				1x
Prolonged fever (>7 days)			1x				1x	1x		
Seizures >12 h after admission						1x		2x	1x	
Low peripheral WBC count				2x	1x	3x	1x	2x	2x	3x
Low CSF WBC count			1x	1x		1x	1x	1x	2x	3x
Low CSF glucose level	2x			1x		1x	1x	1x	2x	6x
High CSF protein level		1x		1x	1x	2x		1x		4x
*S. pneumonia *as causative pathogen	2x		2x	1x						
Young age						1x	1x	2x	1x	1x
*<1 years*						*1x*		*1x*	*1x*	*1x*
*<2 years*							*1x*	*1x*		
Male gender	1x		1x	1x					2x	
Fever >40°C			1x			1x				
Absence of petechiae	1x		1x						1x	

In total, 17 factors were regarded as showing some evidence of importance in the prediction of sequelae or mortality after BM.

- For hearing loss, the factors *S. pneumoniae *as a causative pathogen and a low cerebrospinal fluid (CSF) glucose level showed some evidence of being important (i.e. reported in more than one moderate/high quality study).

- For mortality, coma and seizures were found to be predictive, next to shock, peripheral circulatory failure, severe respiratory distress, a low peripheral white blood cell (WBC) count and a high CSF protein level.

- For neurological sequelae in general, coma, seizures, prolonged fever for at least seven days and a low CSF (WBC) count were considered important risk factors.

Studies reporting on poor outcome, and thereby not differentiating between sequelae or mortality, also reported coma, seizures, shock, a low WBC count both peripheral as well as in CSF and a low CSF glucose level and a high CSF protein level to be important risk factors. Yet they also identified young age (indicated as younger than two years old) and prolonged seizure duration (>12 hours after admission) as important prognostic factors.

When considering all moderate/high quality studies combined, the factors of history of symptoms longer than 48 hours, male gender, fever and absence of petechiae were also found more than once. Although these factors have not been found in more than one study of moderate/high quality for a specific outcome category, they may be important prognostic factors for sequelae or mortality in general.

The 17 identified risk factors were also found in several studies of low quality (see last column of Table 4).

## Discussion

We identified 31 studies in the literature on prognostic factors predicting sequelae or death due to BM in children 0-18 years of age. The included studies have presented a large number of potentially important prognostic factors. Only those factors reported in more than one moderate/high quality study were considered as showing some evidence of being important. These factors included several clinical parameters: coma/impaired consciousness, seizures, shock, peripheral circulatory failure, severe respiratory distress, (prolonged) fever and prolonged duration of seizures, which are all signs of severity during the acute phase of the disease. In addition, the presented factors also included results from diagnostic tests which are performed during admission of the patient in the hospital: low peripheral WBC and low WBC count in CSF, low CSF glucose level and high CSF protein level. These factors are indicators of an acute severe CNS infection and thus are also parameters of severity of the disease.

The presence of these clinical and diagnostic factors in our study demonstrates that severe illness at admission contributes to BM-related mortality and long-term sequelae. In addition, young age was also considered an important prognostic factor. This might be explained by the immature immune status resulting in more severe infections (especially in neonates and children younger than six months) and the developing (and thus more vulnerable) brain of young children. Although it is thought that young children have a higher capability of neurogenesis than older children and adults which leads to better structural repair of brain tissue, and it is known they have a higher plasticity of the brain that allows intact parts to take over functions of damaged areas, early disruption of the developing brain may leads to more functional damage [40-43]. Further, sequelae of meningitis like epilepsy, cerebral palsy and hearing problems can independently cause developmental problems in the young child.

Another prognostic factor which we also demonstrated to be related to severity was the causative pathogen of BM. *S. pneumoniae *seemed to be an important prognostic factor, suggesting a more pathogenic potency of this species in comparison to other bacteria. This has also been found in other studies presenting association or prognostic models in children or adults [3,10,44]. We also found the absence of petechiae to be a prognostic factor. Since petechiae are strongly related with the causative pathogen (occurring mostly in *Neisseria meningitidis *infections, and much less in *S. pneumoniae *meningitis), it supports the finding that *S. pneumoniae *is responsible for a non favorable outcome. In studies of high and moderate quality that reported the absence of petechiae as a risk factor, *S. pneumoniae *was also a prognostic factor of importance. Finally, male gender was found as an important prognostic factor, for which we do not have an explanation. All of these factors might be important to assess in children with BM when trying to identify those at the highest risk for the development of sequelae.

The main concern about the interpretation of the prognostic factors is the fact that due to limited quality of the included studies and heterogeneity of the data it is impossible to perform a meta-analysis and to construct an overall prediction model.

### Limitations

The search strategy was restricted to full report articles published in English, in journals available in the used electronic databases. This might have led to language or publication bias by missing relevant studies.

The quality of studies was assessed using the QUIPS instrument, designed for prognosis studies addressing all common sources of bias. The QUIPS, however, lacks discriminative power. We defined a scoring algorithm for better discrimination of study quality. This scoring algorithm and cutoff points used to qualify the quality of the studies are quite arbitrary. However, all identified prognostic factors found in the included studies are presented in Table 3, allowing readers to draw their own conclusions.

We encountered some problems in interpreting the results of the studies. Only significant prognostic factors of the original studies were presented in our review. However, lack of statistical significance may be due to lack of power. Furthermore, many studies performed only univariate analysis and the presented factors might not have been found significant if multivariate analysis had been performed.

In our overview of prognostic factors we only stratified per type of outcome. We did not compare other subgroups, thereby ignoring the heterogeneity in all other study characteristics (study design, method of analysis, follow-up duration, population, age at infection, pathogen and country of study). We refrained from this since strata would include too few studies of moderate/high quality and too many prognostic factors to discriminate between the groups and draw reliable conclusions.

Finally, due to the limited quality of most studies, and the heterogeneous nature of study characteristics and results, the factors found must only be used with caution.

## Conclusions

Several plausible and important prognostic factors for the prediction of sequelae or mortality after BM in childhood were proposed. Because of the limited quality of most studies and the heterogeneous nature of study characteristics and results, findings must be interpreted critically and the prognostic factors found may be used only with caution. This demonstrates that more high quality prognostic studies on factors related to sequelae or death after BM in childhood are clearly needed.

## Competing interests

The authors declare that they have no competing interests.

## Authors' contributions

RdJ, MvF, MW and CT had primary responsibility for protocol development and writing of the manuscript. RdJ and MW were responsible for the selection and quality assessment of the articles and extraction and analysis of the data. RG substantial contributed to the writing of the manuscript.

All authors read and approved the final manuscript.

## Appendix 1

The 10 most common causative pathogens of BM according to the Netherlands Reference Laboratory for Bacterial Meningitis [38]:

-*Streptococcus pneumoniae*

-*Neisseria meningitidis*

-*Haemophilus influenzae type B (HiB)*

-*Listeria monocytogenes*

-*Escherichia coli*

-*Streptococcus agalactiae (Group B Streptococcus, GBS)*

-*Streptococcus pyogenes*

-*Staphylococcus aureus*

-*Coagulase-negative Staphylococcus (CoNS)*

-*Cryptococcus neoformans*

## Appendix 2: used search strategies for Medline and Embase

### Medline

#### #1 search terms on "Bacterial meningitis"

"Meningitis, Bacterial"[Mh] OR "Meningitis, Bacterial/complications"[Mh] OR "Meningitis, Bacterial/diagnosis"[Mh] OR "Meningitis, Bacterial/epidemiology"[Mh] OR "Meningitis, Bacterial/physiopathology"[Mh] OR "Meningitis, Bacterial/psychology"[Mh] OR "Meningitis, Meningococcal"[Mh] OR "Meningitis, Meningococcal/complications"[Mh] OR "Meningitis, Meningococcal/diagnosis"[Mh] OR "Meningitis, Meningococcal/mortality"[Mh] OR "Meningitis, Pneumococcal"[Mh] OR "Meningitis, Pneumococcal/complications"[Mh] OR "Meningitis, Pneumococcal/diagnosis"[Mh] OR "Meningitis, Pneumococcal/mortality"[Mh] OR "Meningitis, Escherichia coli"[Mh] OR "Meningitis, Escherichia coli/complications"[Mh] OR "Meningitis, Escherichia coli/diagnosis"[Mh] OR "Meningitis, Escherichia coli/mortality"[Mh] OR "Meningitis, Haemophilus"[Mh] OR "Meningitis, Haemophilus/complications"[Mh] OR "Meningitis, Haemophilus/diagnosis"[Mh] OR "Meningitis, Haemophilus/mortality"[Mh] OR "Meningitis, Listeria"[Mh] OR "Meningitis, Listeria/complications"[Mh] OR "Meningitis, Listeria/diagnosis"[Mh] OR "Meningitis, Listeria/mortality"[Mh] OR meningitis[tw] AND (bacterial[tw] OR meningococcal[tw] OR pneumococcal[tw] OR Neisseria[tw] OR meningitides[tw] OR Streptococcus[tw] OR pneumoniae[tw] OR Haemophilus[tw] OR Hib[tw] OR influenzae[tw] OR Listeria[tw] OR monocytogenes[tw] OR Escherichia[tw] OR coli[tw] OR agalactiae[tw] OR pyogenes[tw] OR Staphylococcus[tw] OR aureus[tw] OR Cryptococcus[tw] OR neoformans[tw])

#### #2 search terms on "prognosis"

Morbidity[Mh:noexp] OR mortality[Mh:noexp] OR "cause of death"[Mh] OR survival rate [Mh] OR prognos*[tw] OR predict*[tw] OR course*[tw] OR longitudinal[tw] OR follow-up[tw] OR followup[tw] OR follow up[tw] OR cohort*[tw] OR survival[tw] OR cohort studies[mh] OR follow-up studies[mh]

#### #3 search terms exclusions

("addresses"[Pt] OR "biography"[Pt] OR "case reports"[Pt] OR "comment"[Pt] OR "directory"[Pt] OR "editorial"[Pt] OR "festschrift"[Pt] OR "interview"[Pt] OR "lectures"[Pt] OR "legal cases"[Pt] OR "legislation"[Pt] OR "letter"[Pt] OR "news"[Pt] OR "newspaper article"[Pt] OR "patient education handout"[Pt] OR "popular works"[Pt] OR "congresses"[Pt] OR "consensus development conference"[Pt] OR "consensus development conference, nih"[Pt] OR "practice guideline"[Pt]) NOT ("animals"[Mh Terms] NOT "humans"[Mh Terms])

#### Final search on Bacterial meningitis and prognosis with exclusions

#1 AND #2 NOT #3

### Embase

#### #1 search terms on "Bacterial meningitis"

((('bacterial meningitis'/exp OR 'bacterial meningitis') OR ('epidemic meningitis'/exp OR 'epidemic meningitis')) OR ('meningitis'/de OR 'meningitis') AND (bacterial OR meningococcal OR pneumococcal OR ('neisseria'/de OR 'neisseria') OR meningitides OR ('streptococcus'/de OR 'streptococcus') OR pneumoniae OR ('haemophilus'/de OR 'haemophilus') OR hib OR influenzae OR ('listeria'/de OR 'listeria') OR monocytogenes OR ('escherichia'/de OR 'escherichia') OR coli OR agalactiae OR pyogenes OR ('staphylococcus'/de OR 'staphylococcus') OR aureus OR ('cryptococcus'/de OR 'cryptococcus') OR neoformans)). include text word

#### #2 search terms on "prognosis"

((('morbidity'/de OR 'morbidity') OR ('mortality'/de OR 'mortality') OR ('cause of death'/exp OR 'cause of death') OR ('survival rate'/exp OR 'survival rate') OR ('cohort analysis'/exp OR 'cohort analysis') OR ('follow up'/exp OR 'follow up')) OR prognos* OR predict* OR course* OR cohort* OR longitudinal OR ('follow up'/de OR 'follow up') OR ('followup'/de OR 'followup') OR ('survival'/de OR 'survival')). include text word

#### #3 search terms exclusions

(('literature'/exp OR 'literature'/de) OR ('case report'/exp OR 'case report'/de) OR ('directory'/exp OR 'directory'/de) OR ('editorial'/exp OR 'editorial'/de) OR ('interview'/exp OR 'interview'/de) OR ('medicolegal aspect'/exp OR 'medicolegal aspect'/de) OR ('reading'/exp OR 'reading'/de) OR ('publication'/exp OR 'publication'/de) OR ('patient education'/exp OR 'patient education'/de) OR ('organization'/exp OR 'organization'/de) OR ('consensus development'/exp OR 'consensus development'/de) OR ('practice guideline'/exp OR 'practice guideline'/de)) NOT (('animal'/exp OR 'animal'/de) NOT ('human'/exp OR 'human'/de))

#### Final search Bacterial meningitis and prognosis with exclusions

#1 AND #2 NOT #3

## Pre-publication history

The pre-publication history for this paper can be accessed here:

http://www.biomedcentral.com/1471-2334/10/232/prepub
